# Psychosocial determinants of child cognitive development in sub-Saharan early childhood development centres: a systematic review protocol

**DOI:** 10.1186/s13643-026-03112-1

**Published:** 2026-02-25

**Authors:** Katlego Magdeline Rantho, Mpsanyana Makgahlela, Ronny Mkhonto, Mapula Mothapo, Lesiba Mphela, Peaceful Ntshayintshayi, Winter Seshoka, Livhuwani Muthelo, Samukezi Mrubula-Ngwenya, Tholene Sodi

**Affiliations:** 1 Department of Psychology, University of Limpopo, Polokwane, South Africa; 2 SAMRC-DSI/NRF-UL SARChI Chair: Mental Health and Society, University of Limpopo, Polokwane, South Africa; 3 Department of Nursing, University of Limpopo, Polokwane, South Africa; 4 Department of Educational Studies, University of Limpopo, Polokwane, South Africa; 5 Department of Cultural and Political Studies, University of Limpopo, Polokwane, South Africa; 6 North-West University, COMPRES, Potchefstroom, South Africa

**Keywords:** Children, Cognitive Development, Early childhood development, Psychological determinants, Social determinants

## Abstract

Cognitive development is fundamental in building children’s future learning and adaptive behaviours. Children’s cognitive development may be influenced by an interplay of psychosocial factors, especially in settings such as sub-Saharan Africa, where resources are limited. Despite the significance of early cognitive stimulation on children, there is a paucity of research for understanding the complex interaction of psychosocial determinants of child cognitive development in Early Childhood Development centres, particularly in sub-Saharan Africa. This review aims to synthesise existing literature on the psychosocial determinants of child cognitive development in sub-Saharan African ECDs. This proposed systematic review will be conducted according to the recommendations of the Preferred Reporting Items for Systematic Review and Meta-Analysis Protocols (PRISMA-P). We will search for primary studies conducted using qualitative, quantitative, and mixed methods approaches on global (PubMed, PsycINFO, ProQuest) and regional databases (Sabinet African Journals, Science Direct, African Index Medicus, and sub-Saharan African institutional repositories). The included studies should report on the psychosocial determinants of child cognitive development of children aged 0–6 years registered within sub-Saharan African ECDs and conducted in English between 2014 and 2024. The quality of studies will be assessed using the Mixed Method Appraisal tool, and the data will be analysed using the content analysis method. We envision that the systematic review will enrich discussions on child cognitive development and facilitate the development of interventions aimed at improving child cognitive development in sub-Saharan African ECDs.

**Systematic review registration** PROSPERO CRD42023470844.

## Introduction

Cognitive development concerns how a child learns to think, reason, and use language to adapt and understand their world, and is an important building block towards intelligent behaviours [1]. The quality of the care environment that children are exposed to in their early years has long-term consequences; hence, cognitive stimulation and early school attendance are positively correlated [2, 3]. Childhood development is recognised as a fundamental and universal human right; consequently, the availability of Early Childhood Development (ECD) centres, which offer a formal early childhood programme crucial for the cognitive development of children under 5 years of age [4, 5].

An international study including the United States of America, Europe, and other countries revealed that the long-term effects of early childhood education include a reduced risk of special education, grade retention, cognitive achievement, and scholastic success [6]. In a study conducted by [7], it was noted that children aged 12–24 who were in centre-based care showed more positive cognitive outcomes than those in full-time home care. Biological, psychological, and socioeconomic factors are seen as determinants of cognitive outcomes [8]. Strauß and colleagues also reported that poor parenting practices, poverty, and malnutrition have long been associated with negative cognitive outcomes [9]. Regrettably, sub-Saharan African countries account for nearly 66% of the world's child population under five who do not reach their full cognitive development potential [10]. However, sub-Saharan Africa lacks research focusing particularly on the psychosocial determinants of child cognitive development in early childhood development centres or preschools. Most of the research on the determinants of child cognitive development comes from studies conducted in middle–high-income countries [10, 11].

Compared to children in Europe and America, those in sub-Saharan Africa are specifically subjected to high levels of poverty and malnutrition [10]. Consequently, children from low-income regions are at a high risk of showing deficits in cognitive and social-emotional development [8, 10]. It is proven that, in comparison to their peers from middle-to-high-income countries, they perform poorly on cognitive tests over time [9, 12] while underperforming in school [13]. This undermines the affected children’s prospects, which perpetuates intergenerational poverty [11]. The situation is concerning because efforts to manage or promote child cognitive development are expected to be implemented early in life, especially during the early days of schooling [14]. Another concern has been that, generally, child and adolescent mental health research is sparse in Africa [15]. This suggests that, apart from the lack of research on child cognitive development, in general, child mental health research and services are neglected in the African region [15]. Through a search for empirical studies in the published literature, this review seeks to identify psychosocial determinants of child cognitive development in sub-Saharan African Early Childhood Development Centres. This systematic review will provide a regional perspective given that the sub-Saharan African region is faced with unique socio-economic, cultural, and policy-related factors that affect the cognitive development of children. Moreover, it will guide relevant stakeholders such as policymakers, school governing bodies, mental health practitioners, and teachers on how to promote the cognitive growth of children. To achieve the aim of this study, the researchers developed the following objectives:

## Objectives

- To identify psychosocial factors that influence child cognitive development in sub-Saharan early childhood development centres.
- To determine the relationship between psychosocial factors and the cognitive development of children in sub-Saharan early childhood development centres.
- To assess how cultural, environmental, and economic factors affect the cognitive development of children in sub-Saharan early childhood development centres.

## Methods

The researchers searched for systematic reviews that were similar and related to their topic on the International Prospective Register of Systematic Reviews (PROSPERO) and Cochrane libraries to ensure that they do not replicate information and reduce research waste [16]. To the best knowledge of the researchers, this review will be the first to synthesise the psycho-social determinants of child cognitive development within the South African Early Childhood Development Centre. According to [16] researchers, researchers must register their systematic reviews and record the procedures that they have followed to ensure transparency and eliminate research bias. This protocol will be reported on the Preferred Reporting Items for Systematic Review and Meta-Analysis Protocols (PRISMA-P). The completed review of this protocol will use the Preferred Reporting Items for Systematic Review and Meta-Analysis for reporting purposes.

### Eligibility criteria

Table 1 below outlines the summary of inclusion and exclusion criteria that will be used to screen articles for potential inclusion in the study.

**Table 1:** Summary of exclusion and inclusion criteria

Criterion	Inclusion	Exclusion
*Participants*	• Studies conducted on children from 0 to 6 years registered in sub-Saharan African Early Childhood Development Centre	Children, caregivers and stakeholders who are not involved in the Early Childhood Development Centre
	• Studies conducted on caregivers of children registered in Early Childhood Development Centres across sub-Saharan Africa	
	• Studies conducted on/with stakeholders involved in sub-Saharan African Early Childhood Development Centre	
*Study design*	• Primary studies that were conducted using qualitative, quantitative and mixed methods approaches in sub-Saharan Africa	Protocols, review studies and government documents will be excluded
	• Clinical and non-clinical studies relating to the psycho-social determinants of child cognitive development in sub-Saharan African ECDs	
	• Empirical grey literature using qualitative, quantitative and mixed method approaches	
*Setting*	Studies conducted within sub-Saharan Africa on Early Childhood Development centres	Studies conducted outside of sub-Saharan Africa
*Language*	Studies conducted in English	Studies conducted in languages other than English
*Period*	2012–2025	Studies conducted before 2012 and after 2025

### Outcomes

The outcome of the study is to identify specific psycho-social factors that significantly impact child cognitive development in sub-Saharan African Early Childhood Development centres. Specifically, the researchers anticipate that the topic will cover primary cognitive measures such as language development, problem-solving skills, memory, and attention, and secondary measures such as social and emotional development, school readiness, and behavioural outcomes. Furthermore, the topic should provide insight into the impact of psycho-social factors on the overall development of children across different geographical regions and demographic groups in sub-Saharan Africa.

### Types of study

The systematic review will include primary studies such as cross-sectional studies, pilot studies, randomised controlled trials, quasi-experimental studies, cohort studies, case-control studies, qualitative and mixed-method studies, as well as empirical grey literature. These studies should have been conducted on the psycho-social determinants of child cognitive development in South African ECDs. The review will exclude protocols, review studies, government documents, and studies that have scored low on the appraisal tool. The included studies should be in the English language, as researchers aim to gather enough data on the topic under study. Restricting studies to those conducted using English may result in the exclusion of non-English language studies, particularly those conducted in Francophone and Lusophone sub-Saharan African countries, which may introduce bias. While this may limit the comprehensiveness of the review, the decision is based on practical constraints such as the English proficiency of all the members of the review and the lack of access to resources and translation services that will assist them in interpreting non-English studies. This limitation is acknowledged and will be considered in the interpretation of the review findings. The review will select studies conducted between 2012 and 2025. The authors selected this period because it aligns with the key global policy developments, which emphasise the significance of appropriate early childhood development. This includes the World Health Organisation initiatives on integrated ECD interventions (World Health Organisation, [17]) and the adoption of Sustainable Development Goals in 2015, particularly Section 4.2, which emphasises quality access to early childhood development care and education (United Nations, [18]).

### Participants and study setting

Prospective eligible studies must be related to the psycho-social determinants of child cognitive development within the Early Childhood Development Centres**.** These studies should have been conducted on children attending ECDs aged 0–6 years. The review will also include studies conducted on caregivers of children in ECDs and stakeholders involved in ECDs.

### Information sources

The reviewers will source articles from global databases (PubMed, ProQuest, PsycINFO, and EBSCOhost) and regional databases (Sabinet African Journal, Science Direct, African Index Medicus, and sub-Saharan African institutional repositories). The reviewers will perform a backward search by checking the references on eligible studies and a forward search by using the “related articles” option found through Google Scholar. We will also go through published protocols and reviews to get primary studies related to our study topic and contact the authors of locked articles to check if they meet our eligibility criteria.

### Search strategy and process

The search technique for this systematic review protocol will include a combination of keywords, Boolean logical operators, truncations, and Medical Subject Headings (MeSH) terms as appropriate for and relevant to each selected database. To ensure a complete geographic scope, a search filter will comprise the articles conducted in countries within the sub-Saharan African region, expressed in English. This strategy seeks to gather a wide range of literature on psycho-social determinants of child cognitive development in the sub-Saharan African Early Childhood Development Centre. The researchers will use the search strategy (“Psychosocial determinants” OR “Social determinants” OR “Environmental determinants” AND “Child Cognitive Development” OR “Cognitive Skills” OR “learning outcomes” OR “Mental development” AND “Early Childhood Development” OR “Early Childhood Education” AND “sub-Saharan Africa” Each of the selected databases will be searched separately, and the results of the searches will be merged before duplicates are eliminated. The Statement for Reporting Literature Searches in Systematic Reviews guidelines will be utilised to guide the reporting of the search process [19].

### Study records

#### Data management

The PRISMA-2020 flow diagram guidelines [20] will be followed as part of the reporting process. Records will be stored in EndNote 21 to keep track of the articles retrieved from numerous databases. This will entail removing duplicates, screening based on titles and abstracts, and retrieving the full text of eligible studies.

#### Selection

All reviewers will undertake a thorough screening of titles and abstracts for the identified studies, using pre-defined inclusion and exclusion criteria. Following the title and abstract screening phase, studies considered to be potentially eligible will undergo full-text review, which will be performed by four selected reviewers. Then, one reviewer will be responsible for mediating and settling discrepancies among the reviewers through consensus to ensure the inclusion of studies is accurate. In cases where further information is required, reviewers will consult published protocols and related online supplemental materials (if available) from eligible studies. If a study is not available online, the authors will be contacted via email correspondence to request any missing or supplemental relevant information.

### Data collection process and data items

Relevant studies identified from the selected databases will be exported to the EndNote 21 reference manager for screening and recording purposes. Before commencing with title and abstract screening, one reviewer (KMR) will remove duplicates on the reference manager and equally divide the studies among five reviewers (RM, MTM, LM, WS, and SM) to screen them using the study title and abstract. In the case where conflicts emerge, the selected reviewers will resolve them through discussion in the presence of a seventh reviewer (MM) to reach a consensus. Upon the completion of the T and A screening, the PDF articles that meet the inclusion criteria will be extracted and appraised by two reviewers (KMR and MM) using the Mixed Method Appraisal Tool (MMAT). Then, data extraction will commence, wherein four reviewers (KMR, PN, MM, and LM) will extract data using a predesigned extraction form. The following data will be extracted: author, year of publication, country, study design, sample characteristics (sampling method, sample size, gender ratio, mean age), the domain of cognitive development reported (knowledge and prevention, recognition, personality), key findings, and study quality rating. Upon having conflicts, one reviewer (TS) will be appointed to resolve conflicts.

### Quality and risk of bias in individual studies

The authors will appraise the methodological quality of included studies using the Mixed Methods Appraisal Tool (MMAT), criteria for qualitative, quantitative, and mixed-method studies. Two authors (KMR and MM) will independently perform this activity to reach a consensus on which studies to include. Should they experience disagreements, another author (TS) will resolve disputes. For risk of bias, the Newcastle–Ottawa Scale (NOS) will be used for observational studies [21]. The scores on NOS range from 0 to 9, wherein points 7–9 indicate low risk of bias, 4–6 indicate moderate risk of bias, and 0–3 indicate high risk of bias. If there are any randomised control trials, the Cochrane Risk of Bias 2 (RoB 2) will be used [22]. The methods used for assessment will not be used as exclusion criteria but will guide the sensitivity analysis. The review will transparently report on the limitations of the risk of bias tools and their application.

### Data synthesis

The data extracted from eligible studies will be synthesised using the narrative synthesis method, complemented by quantitative approaches where necessary, depending on the heterogeneity across studies. The analysis process will follow the three-step approach to narrative synthesis by Petticrew and Roberts to synthesise the evidence drawn from the included studies: (i) organizing the description of the studies into logical categories; (ii) analysing the findings within each of the categories; and (iii) synthesizing the findings across all included studies [23]. Given the nature of our topic, we expect a limited scope of meta-analysis because of the anticipated high variability in studies. However, we will employ the Jamovi programming software package (V.1.8.1) to describe statistics, subgroup comparisons, present visual data, or any other quantitative analysis that may be feasible.

Given the anticipated heterogeneity from various tools and measures for developmental outcomes, such as observational checklists and screening tools, the researchers will conduct a meaningful quantitative synthesis wherein outcomes are standardised. To compare continuous outcomes, standardised mean differences will be calculated with 95% confidence intervals to ensure that effect sizes are consistent across various scales. Meanwhile, the categorical data will be calculated and synthesised using odds ratios or risk ratios where appropriate. The heterogeneity will be assessed using the *I*^2^ statistic, and meta-analysis will be conducted using random-effect models to factor in the differences in the study population, settings, and tools. In addition, the researchers will adjust effect estimates in cases where clustering has not been accounted for and for studies which have used clustered sampling designs using the available intra-class correlation coefficients and average cluster sizes. Moreover, the subgroup and sensitivity analysis will explore the differences using elements such as the geographic location, child age groups, type of psychological determinant, and socioeconomic status. The visual data will be presented in the form of tables and figures or even forest plots, where appropriate, to interpret the data in a clear manner [23].

## Discussion

This review aims to synthesise the psychosocial factors influencing child cognitive development in the Early Childhood Development Centres, highlighting a notable gap in research within Sub-Saharan Africa regarding these determinants. The completion of this review is intended to enrich discussions on child development in the region and propose potential solutions. It emphasises that childhood development is a fundamental human right that should not be overlooked. In comparison to Western and Northern countries, children in Sub-Saharan Africa face significant challenges, including high levels of poverty and malnutrition [10]. Thus, the authors are focused on identifying strategies to enhance child development in this region. The review will detail the target population through a critical analysis of studies addressing the psychosocial determinants of cognitive development in the Early Childhood Development Centre in Sub-Saharan Africa, irrespective of age or gender. The authors expect the findings to highlight primary cognitive measures such as language development, problem-solving skills, memory, and attention, along with secondary outcomes including social and emotional development, school readiness, and behavioral results in the African context.

## Data Availability

Data sharing is not applicable yet since this is a systematic review protocol, and no data have been accessed yet.
